# The Evolving Role of Bispecific Antibodies in Diffuse Large B-Cell Lymphoma

**DOI:** 10.3390/jpm14070666

**Published:** 2024-06-21

**Authors:** Khalil Saleh, Rita Khoury, Nadine Khalife, Claude Chahine, Rebecca Ibrahim, Zamzam Tikriti, Axel Le Cesne

**Affiliations:** 1 International Department, Gustave Roussy Cancer Campus, 94800 Villejuif, France; rita.khoury011@gmail.com (R.K.); claude.chahine@gustaveroussy.fr (C.C.); rebecca.ibrahim@gustaveroussy.fr (R.I.); zamzam.tikriti@gustaveroussy.fr (Z.T.); axel.lecesne@gustaveroussy.fr (A.L.C.); 2 Department of Head and Neck Oncology, Gustave Roussy Cancer Campus, 94800 Villejuif, France; khalife.nadine@live.com

**Keywords:** diffuse large B-cell lymphoma, epcoritamab, glofitamab, mosunetusumab, odronextamab, bispecific antibodies, polatuzumab

## Abstract

The advent of targeted therapies such as monoclonal antibodies, adoptive T-cell therapies, and antibody–drug conjugates (ADCs) dramatically changed the treatment landscape of diffuse large B-cell lymphoma (DLBCL) over the last two decades. Rituximab was the first one approved. Chimeric antigen receptor T-cells are currently approved as second-line treatment in patients with DLBCL refractory to first-line chemo-immunotherapy. Polatuzumab, a CD79b-targeting ADC, is approved as first-line treatment in high-risk patients in combination with chemo-immunotherapy. Bispecific antibodies (BsAbs) are a novel category of drugs that are also changing the treatment paradigm of patients with DLBCL. They are engineered to bind to two different targets at the same time. To date, two BsAbs (glofitamab and epcoritamab) are approved as monotherapy in third-line treatment in DLBCL. Combination strategies with chemotherapy, immunotherapy, and ADCs are currently under investigation with encouraging results in first-line or subsequent lines of treatment. In the following review, we focus on the structure of BsAbs, the mechanism of action, clinical efficacy, and the mechanisms of resistance to BsAbs.

## 1. Introduction

The treatment paradigm of diffuse large B-cell lymphoma (DLBCL) has drastically changed during the past few decades. The introduction of rituximab, an anti-CD20 chimeric monoclonal antibody, revolutionized the management of DLBCL when combined with chemotherapy [1,2]. Thus, systemic immunochemotherapy regimens such as R-CHOP (rituximab, cyclophosphamide, doxorubicin, vincristine, and prednisone) or R-ACVBP (rituximab, doxorubicin, cyclophosphamide, vindesine, bleomycin, and prednisone) remain the first-line treatment of DLBCL [1,3]. The role of autologous stem cell transplantation (ASCT) in first remission is controversial [4]. However, ASCT was the standard of care preceded by salvage chemotherapy in relapsed and/or refractory (R/R) DLBCL. The development of immunotherapy continues to change the treatment paradigm of patients with DLBCL and to improve the results in R/R disease. Autologous chimeric antigen receptor (CAR) T-cells were developed for the management of R/R DLBCL. The Food and Drug Administration (FDA) initially approved three CAR T-cells targeting CD19 for the treatment of patients with R/R DLBCL after the failure of two previous treatment lines. The three CAR T-cells were axicabtagene ciloleucel, tisagenlecleucel, and tisocabtagene maraleucel and were associated with an overall response rate (ORR) ranging from 52% to 82% [5,6,7]. More recently, axicabtagene ciloleucel and lisocabtagene maraleucel were also approved by the FDA as second-line treatment in patients with R/R DLBCL based on the ZUMA-7 and TRANSFROM phase III trials, respectively. These trials compared CAR T-cells with ASCT [8,9]. Another option for R/R disease is the advent of antibody–drug conjugates (ADCs). ADCs are monoclonal antibodies that selectively bind to a target antigen and deliver cytotoxic agents through internalization. Polatuzumab vedotin is an anti-CD79b ADC, also coupled to monomethyl auristatin E (MMAE). The combination of polatuzumab vedotin with bendamustine and rituximab (BR) showed a superior complete response (CR) rate and reduced the risk of death in comparison with BR in patients with R/R disease [10]. Moreover, the POLARIX (polatuzumab plus rituximab) phase III trial compared polatuzumab vedotin + R-CHP (rituximab, cyclophosphamide, doxorubicin, and prednisone) versus R-CHOP in patients with newly diagnosed, previously untreated DLBCL. It showed a 36% reduction in the risk of progression, relapse, or death with a 7.7% improvement in 2-year progression-free survival (PFS) with polatuzumab vedotin + R-CHP compared to R-CHOP in patients with intermediate- to high-risk disease [11]. The POLARIX trial led to the approval of polatuzumab vedotin in the first-line setting. More options in R/R disease include tafasitamab, a humanized, Fc-modified, CD-19-targeting monoclonal antibody that was approved in combination with lenalidomide in patients with R/R DLBCL based on the results of the L-MIND phase II trial [12]. Given the activity of T-cell-based therapies, a novel class of monoclonal antibodies known as bispecific antibodies (BsAbs) has been developed. Several agents are under clinical development and investigation such as epcoritamab, glofitamab, mosunetuzumab, and odronextamab. The FDA approved epcoritamab and glofitamab for the management of R/R DLBCL. In this review, we summarize the multiple BsAbs under evaluation with their structure and mechanism of action. We also discuss the clinical benefit of these agents as well as their place in the therapeutic arsenal of DLBCL in comparison with autologous CAR T-cells and the possible mechanism of resistance.

## 2. Structure and Mechanism of Action of Bispecific Antibodies

BsAbs are genetically produced to recognize and bind to two different targets (antigens or epitopes) simultaneously. Two monospecific antigen-binding regions, which freely recognize their respective precise target, are combined together to make a single antibody-derived molecule that acts as a bridge for these two antigens. BsAbs can redirect cytotoxic natural killer (NK) cells or T-cells near to tumor cells, contributing to enhanced cytotoxicity and allowing for the concomitant inhibition of two different signaling pathways, and may enhance binding specificity via the interaction with two cell-exposed antigens [13,14].

BsAbs can be classified as two different groups: immunoglobulin G (IgG)-like proteins and non-IgG-like proteins. IgG-like proteins are long-lived BsAbs (>150 kDa) that contain a human immunoglobulin constant region, fragment crystallizable (Fc) region, and classic antibody backbone with two fragment antigen-binding (Fab) sites. the two Fab arms bind different targets. They usually have longer half-lives and increased affinity and then can survive up to several days and enhance biological activity. These antibodies conserve the Fc-mediated immune effector functions such as antibody-dependent cellular phagocytosis (ADCP), complement-dependent cytotoxicity (CDC), and antibody-dependent cell-mediated cytotoxicity (ADCC). The Fc fragment contributes to improved solubility and stability and to facilitated purification [15]. Non-IgG-like proteins are smaller proteins and mainly consist of single-chain variable fragments (scFvs). They are generated using the fusion of variable domains of the IgG heavy chain and light chain through a flexible linker [16]. The small weight leads to increased tissue penetration and is cleared within a few hours. Consequently, their therapeutic effects are limited to antigen binding. These molecules are easy to engineer and have low immunogenicity [17].

The majority of BsAbs in development for DLBCL have a full-length, IgG-like structure. The construction of BsAbs is complex and requires different steps. Most commonly, a quadroma is generated by the fusion of two pre-established hybridomas that express two different monoclonal antibodies. The quadroma expresses parental light chains and heavy chains. The random fusion of several light and heavy chains might contribute to several distinct products. The triomab, a functional bispecific antibody, represents a small proportion of the final product and is associated with a high rate of heavy chain homodimerization and light chain mispairing. Several methods were used to overcome this issue. Knobs-into-holes is one of these methods and consists of substituting a smaller amino acid with a larger amino acid (T336Y) in the CH3 domain of an antibody to generate a knob structure and at the same time replacing a larger amino acid on the corresponding CH3 with a smaller one to form a hole structure (Y407T). The knobs-into-holes technique overcomes heavy chain homodimerization but might be limited by light chain mispairing. The CrossMab technology can solve the mispairing of light chains by switching CH1 and VL domains to construct modified heavy and light chains. In the CrossMab2:1 technique, an extra Fab can be added to create a trivalent product. Producing BsAbs using the common light chains technique that consists of the same light chains interacting with two different heavy chains can also overcome light chain mispairing but can limit binding specificities and is not applicable to all BsAbs. Multiple other technologies have been used to produce IgG-like BsAbs such as controlled Fab-arm exchange where two parental antibodies are expressed separately and mixed thereafter. The best recombinant candidate called DuoBody is selected according to its capacity to bind both epitopes and induce cytotoxicity as well as to show high purity [18,19]. The production of non-IgG-like BsAbs is in general more direct. BiTe (bispecific T-cell engager) and DART (dual-affinity re-targeting antibody) are the most frequent models. BiTe consists of a single chain containing the four variable domains of heavy and light chains. However, DART consists of two chains containing variable domains of heavy and light chains and stabilized by a C-terminal disulfide bridge [20].

## 3. Clinical Efficacy of Bispecific Antibodies in DLBCL

### 3.1. Bispecific Antibodies as Single Agent

Blinatumomab (MT103), a bispecific anti-CD19 CD3 T-cell engager, was the first BsAb that entered the clinical arena and obtained FDA approval for the management of R/R B-cell acute lymphoblastic leukemia. Blinatumomab was associated with encouraging activity with durable benefit (median duration of response of 404 days) in a phase I trial of patients with R/R non-Hodgkin lymphoma (NHL) including patients with DLBCL. However, it was associated with significant rates of cytokine release syndrome (CRS) and immune effector cell-associated syndrome (ICANS) (22% of patients with grade 3 ICANS) [21]. In the subsequent phase II trial of blinatumomab in heavily pretreated R/R DLBCL with modified administration strategies and prophylactic dexamethasone, the objective response rate (ORR) was 43%, including 19% CR. The median duration of response (DOR) was 11.6 months [22]. The clinical usage of blinatumomab in DLBCL has been limited by the increased incidence of CRS, neurological toxicities, and the unpractical administration.

Mosunetuzumab (BTCT4465A) is the first-in-class, fully humanized IgG1 BsAb targeting CD20 (cluster of differentiation 20), a B lymphocyte surface antigen, and CD3, expressed exclusively by mature T-cells. Mosunetuzumab was developed using knob-in-hole engineering. It has been produced in both subcutaneous and intravenous formulations. The response rates of the two formulations were similar [23,24]. Based on the results of a phase II trial, the FDA granted an accelerated approval for mosunetuzumab for the management of R/R follicular lymphoma (FL) after two or more lines of systemic treatment in December 2022. The ORR was 80%, including 60% CR, and the median DOR was 22.8 months [25]. In the single-arm expansion cohort of R/R DLBCL who progressed at least after two previous lines, mosunetuzumab was associated with an ORR of 42% (35/88 patients), and the CR rate was 24% (21/88 patients). The median PFS was 3.2 months. CRS was the most common adverse event (26% of patients) consisting of a manageable safety profile [26]. In a phase I study evaluating mosunetuzumab in B-cell NHL, mosunetuzumab was associated with an ORR of 35% in aggressive NHL (CR was 19.4%) [27]. Single-agent mosunetuzumab was also investigated in the GO40554 phase I/II trial in elderly patients with previously untreated DLBCL unfit to standard chemo-immunotherapy. Overall, 54 patients were included as of June 2022. The median age was 83 years with a range of 65 to 100. At a median follow-up of 23.3 months, the ORR was 56% (30/54 patients) including 43% CR (23/54 patients) across treatment groups. The 12-month PFS rate was 39%. The median duration of CR was 15.8 months [28]. Overall, mosunetuzumab showed modest activity in aggressive lymphomas leading to the development of combination strategies in these patients.

Epcoritamab (Gen3013) is a subcutaneous full-length IgG1 BsAb engineered with bivalency for CD20 to allow for the co-binding of rituximab and/or obinutuzumab. The results of the phase I trial were recently published by Hutchings and colleagues. A total of 73 patients with R/R B-cell NHL were enrolled, and 68 patients received escalated full doses (0.0128–60 mg). There were no dose-limiting toxicities, and the maximum tolerated dose was not reached. Moreover, the recommended phase II dose was fixed as 48 mg. The ORR in patients with R/R DLBCL at full doses of 12 to 60 mg was 68%, including 45% CR. In addition, the ORR at 48 mg was 88%, including 38% CR. The most frequent adverse events (AEs) were pyrexia (69%), CRS (59%), and injection site reactions (47%). There was no grade ≥ 3 CRS, and no discontinuations occurred due to treatment-related adverse events (TRAEs) [29]. In a phase I/II trial of patients with R/R large B-cell lymphoma (EPCORE NHL-1), epcoritamab in the expansion cohort was associated with an ORR of 63% (99/157 patients) with 39% CR (61/157 patients). It is to be noted that response rates were similar in CAR T-cell-naïve patients and CAR T-cell-exposed patients (ORR 69% vs. 54%). The median PFS was 4.4 months; the median overall survival (OS) was not reached. The most frequent TRAEs were CRS in 49.7% of patients, fever (23.6%), and fatigue (22.9%), and ICANS occurred in 6.4% of patients [30]. Based on the results of EPCORE NHL-1 (epcoritamab in non-Hodgkin lymphoma-1), the FDA granted an accelerated approval to epcoritamab for the management of R/R DLBCL and high-grade B-cell lymphoma that failed at least two lines of treatment in May 2023. Epcoritamab is currently under investigation in phase III in comparison with physicians’ choice in patients with R/R DLBCL ineligible for curative therapy. Table 1 summarizes the major clinical trials evaluating BsAbs as monotherapy for the treatment of DLBCL.

Glofitamab (RG6026) is a BsAb targeting CD20 and CD3 and was evaluated in a phase I/II trial (NP30179) of patients with R/R DLBCL who were treated with at least two previous lines. The phase I trial included 171 patients. The ORR was 53.8%, including 36.8% CR in patients treated with the recommended phase II dose [31]. In the phase II part of a phase I–II trial, 155 patients were enrolled. All patients received pretreatment with obinutuzumab to deplete B-cells. The 12-month PFS rate was 37%. At a median follow-up of 12.6 months, 39% of patients had a CR. The median PFS and OS were 4.9 months and 11.5 months, respectively. Interestingly, 33% of patients already failed CAR T-cell therapy, and the CR rate in this subgroup was 35%. CRS was the most common AE that was observed in 63% of patients. Moreover, 62% of patients presented grade ≥ 3 AE (4% and 3% with CRS and neurologic events, respectively). Nearly 9% of patients discontinued glofitamab due to AEs [32]. Based on these results, the FDA granted an accelerated approval for the management of patients with R/R DLBCL or large B-cell lymphoma who failed at least two lines of systemic treatment. In real-world data of 43 patients with R/R DLBCL who received glofitamab with no strict selection criteria, the ORR was 37%, including 21% CR and 16% partial response (PR). The median number of previous lines was four. The median PFS and OS were 3.3 months and 8.8 months, respectively [33].

Odronextamab (REGN1979) is a fully human IgG4-based BsAb that targets CD3 and CD20. Bannerji and colleagues reported the results of a phase I trial evaluating odronextamab in patients with R/R CD20-positive NHL. Overall, 145 heavily pretreated patients were included. The ORR of the entire cohort was 51%. In FL, the ORR was 91% (29/32 patients), including 72% CR among patients who were treated with odronextamab doses of 5 mg and higher. In patients with DLBCL without prior exposure to CAR T-cell therapy and who received odronextamab doses of 80 mg or higher, the ORR was 53%, and all responses were CR (8/15 patients). However, in patients who previously failed CAR T-cell therapy, the ORR was 33% (10/30 patients), including 27% CR. Regarding the safety profile, the most frequent grade ≥ 3 TRAEs were anemia (25%), lymphopenia (19%), hypophosphatemia (19%), neutropenia (19%), and thrombocytopenia (14%). Furthermore, serious TEAEs were reported in 61% of patients (89/145 patients), and the most common were CRS (28%), fever (8%), and pneumonia (6%) [34]. The ELM-2 phase II trial evaluated odronextamab in patients with R/R NHL (NCT03888105). The results of the cohort that included patients with R/R DLBCL were presented at the 2022 American Society of Hematology annual meeting. The recommended phase II dose was 160 mg weekly, and odronextamab was administered with a step-up regimen of 1 mg split over cycle 1 day 1 and 2, 20 mg split over cycle 1 day 8 and 9, and then 160 mg full dose on cycle 1 day 15. Overall, 121 patients with R/R DLBCL were evaluable for safety and 90 patients evaluable for efficacy. The ORR was 53% (48/90 patients), including 37% CR (33/90 patients). The median duration of CR was 17.9 months after a median follow-up of 26.2 months. It is to be noticed that TEAEs were reported in 97% of patients (117/121), and the most frequent were CRS (53%), pyrexia (41%), and anemia (34%). ICANS occurred in only 4% of patients and was low-grade. Grade 5 TEAEs occurred in 2% of patients [35,36]. Based on these results, the FDA granted priority review seeking the approval of odronextamab for the treatment of adult patients with R/R DLBCL who have progressed after at least two prior systemic lines in September 2023.

### 3.2. Bispecific Antibodies in Combination

Preclinical data confirmed that surviving T-cells following treatment with immunochemotherapy can also be activated against lymphoma cells when stimulated by a BsAb [37]. Moreover, T-cells can also be activated and redirected to kill lymphoma cells even in the presence of T-cell cytotoxic agents such as high-dose corticosteroids or cyclophosphamide [38,39]. Furthermore, the combination of a BsAb with an anti-CD20 monoclonal antibody appears feasible and active due to partially overlapping epitopes, the absence of competition for Fc receptors for IgG (FcyR), and the high target cell killing even at low occupancy rates [40].

#### 3.2.1. Combination Strategies in R/R DLBCL

In a phase Ib/II GO40516 trial, mosunetuzumab was evaluated in combination with polatuzumab in patients with R/R aggressive B-cell lymphoma. At a median follow-up of 5.3 months, 60 patients were treated. Older patients (>65 years) had a numerically higher ORR (72% vs. 54%) and CR rate (56% vs. 38%) than younger patients (<65 years). The rates of CRS and AEs were comparable across age groups [41].

Epcoritamab was combined with R-DHAX/C (rituximab, dexamethasone, high-dose aracytine, oxaliplatin/carboplatin) in arm 4 of the EPCORE NHL-2 phase I/II trial in patients with R/R DLBCL and eligible for ASCT. The combination was associated with promising activity with an ORR of 83% (19/23 patients), including 61% complete metabolic response (CMR). Interestingly, among patients who underwent ASCT, the ORR was 100% (11/11 patients), including 86% CMR. The combination had a manageable safety profile [42]. Moreover, in arm 5 of ECPORE NHL-2, epcoritamab was combined with gemcitabine and oxaliplatin in patients with R/R DLBCL ineligible for ASCT. The ORR was 92% (23/25 patients), including 60% CMR. The most frequent TRAE was CRS that occurred in 70% of patients, all were grade 1–2 [43]. More recently, the results of arm 1 of EPCORE NHL-5 evaluating epcoritamab in combination with lenalidomide in patients with R/R DLBCL were reported at the 2023 American Society of Hematology annual meeting. The combination showed encouraging antitumor activity with an ORR of 75% (18/24 patients), including 58% CR (14/24 patients). The median time to first response was 1.8 months. The combination showed a tolerable safety profile with neutropenia as the most frequent grade 3 or more TEAE (58%) followed by anemia and thrombocytopenia (15%) [44].

Glofitamab was evaluated in combination with polatuzumab vedotin in patients with R/R DLBCL in a phase I/II trial and was associated with encouraging activity (ORR of 73%) and a tolerable safety profile with CRS and neurological adverse events limited to grade 1–2 [45]. Moreover, glofitamab in combination with englumafusp alpha, an antibody-like fusion protein targeting CD19 on B-cells and 4-1 BB on T-cells, was evaluated in a phase I clinical trial. In fact, englumafusp alpha strongly stimulates T-cells via 4-1 BB agonism, and glofitamab stimulates 4-1 BB upregulation on immune cells. Overall, 71 patients with R/R B-cell lymphoma were enrolled in the clinical trial including 46 patients with DLBCL. The ORR and CR rate were 67% and 39%, respectively, in patients with DLBCL. Grade  ≥  3 AEs were observed to occur in 57.2% patients [46].

#### 3.2.2. Combinations in Previously Untreated DLBCL

Mosunetuzumab was also evaluated in combination with CHOP in patients with newly diagnosed untreated DLBCL in the ongoing GO40515 phase I–II trial. A total of 36 patients with newly diagnosed DLBCL were enrolled. The ORR was 96%, and the CR rate was 85%. Grade ≥ 3 AEs occurred in 86% of patients and serious adverse events (SAEs) in 44% of patients. CRS occurred in 53% of patients; all of them were grade 1 or 2 and recovered with supportive care [47]. Mosunetuzumab in combination with polatuzumab showed preliminary efficacy in elderly and/or frail patients with previously untreated DLBCL. The ORR and CR rates were 80% and 61%, respectively. The combination was also associated with a manageable safety profile with neutropenia as the most frequent grade 3 or higher AE [48].

In newly diagnosed high-risk DLBCL, epcoritamab is also under evaluation in combination with R-CHOP in phase I/II EPCORE NHL-2. The combination showed promising activity with an ORR of 100% (46/46 evaluable patients), and the CMR was 76%. It was also associated with a manageable safety profile, CRS was reported in 60%, mostly of low-grade (57%), and only one patient presented a grade II ICANS [49]. EPCORE DLBCL-2 is an ongoing phase III trial comparing epcoritamab + R-CHOP versus R-CHOP alone (NCT05578976). Table 2 summarizes major clinical trials of BsAbs in combination with chemotherapy and/or immunotherapy in patients with DLBCL.

Glofitamab is under investigation in a phase Ib trial in combination with R-CHOP in patients with newly diagnosed untreated DLBCL. The ORR was 93.5% (43/46 evaluable patients), including 76.1% CR. The authors reported grade 3 or higher AEs in 23.2% of patients. There was no reported grade 3 to 5 CRS [50]. Moreover, Dickinson and colleagues reported at the 2023 American Society of Clinical Oncology annual meeting the results of a phase Ib trial of patients with previously untreated DLBCL who received glofitamab + pola-R-CHP. Overall, 24 patients were enrolled, and evaluation for efficacy was available for 17 patients. The complete metabolic response (CMR) rate was 76.5% (13/17 patients), and the ORR was 100% at a median follow-up of 5.1 months. Grade 3 or more AEs and AEs related to glofitamab were reported in 63% and 38% of patients, respectively [51].

## 4. Safety Profile and Toxicity of BsAbs

The safety profile and toxicity of BsAbs were similar across prospective trials, and the majority of adverse events were manageable with rare discontinuations and/or interruptions of BsAbs. Adverse events were mostly related to T-cell overactivation. CRS was the most common AE reported in 15% to 80% of patients treated with BsAbs and notably related to the drug, route of administration, dosing, and the schedule of administration. CRS occurs 0.5 to 2 days following BsAb administration and persists for 1.5 to 3 days, mainly during the first cycle of treatment. Most reported CRS were grade 1 to 2 using the grading initially developed for CAR T-cell therapy [52,53]. Neurological toxicity, also related to T-cell overactivation, was reported following the administration of BsAbs but was rare, generally mild, and self-limited within hours of onset in the clinical trials in contrast immune effector cell-associated neurotoxicity following CAR T-cell therapy [27,29,31,34]. The step-up dosing strategy was used to prevent or minimize the severity of AEs related to T-cell overactivation. Other preventive measures were adopted such as the use of prophylactic corticosteroids at the beginning of the treatment, inpatient administration, and in the case of glofitamab, a single dose of obinutuzumab [27,29,31,34,54,55]. The most common adverse events not related to T-cell overactivation were neutropenia (15 to 33%), fatigue (18% to 42%), hypophosphatemia (13% to 29%), anemia (19% to 38%), and diarrhea (15% to 26%) [56].

Interestingly, it seems that the addition of BsAbs to standard chemotherapy or immunotherapy resulted in higher responses and did not produce new adverse safety signals.

## 5. Mechanisms of Resistance to Bispecific Antibodies

The promising activity of bispecific antibodies is counterbalanced by the emergence of resistance. Three categories of potential mechanisms of resistance to BsAbs have been evoked. The first mechanism is a tumor cell-intrinsic one. In fact, selective inhibition may contribute to the activation of several programs leading to immune escape. It was reported that treatment with CD20-targeting monoclonal antibodies led to the loss of target antigen CD20 [57,58,59]. Emerging data suggest that patients with progressive or recurrent disease after treatment with BsAbs presented a loss of CD20. However, the baseline CD20 expression level is not clearly associated with response to these agents [60]. Schuster and colleagues reported that during treatment with mosunetuzumab, the loss of CD20 expression was one mechanism of acquired resistance, but CD20 expression was also present in patients with progressive disease suggesting the presence of alternative mechanisms of acquired resistance [61]. In a recent report, Duell and colleagues demonstrated that truncating mutations in the MS4A1 gene encoding CD20 leading to antigen loss were present using the whole exome sequencing technique in 80% of patients who progressed on CD20-targeting BsAbs [62]. Moreover, molecular analysis conducted on biopsies of patients with R/R NHL and treated with glofitamab showed that tumor cell-intrinsic factors such as a higher frequency of TP53 mutations or overactivated MYC signaling were associated with de novo or acquired resistance to glofitamab [63]. Interestingly, TP53 mutations and MYC amplification are associated with resistance to immunotherapy including CAR T-cell therapy [56]. The second mechanism of resistance is a T-cell-intrinsic mechanism related to acquired T-cell dysfunction among the tumor microenvironment (TME). It includes the activation of regulatory T-cells, downregulation of the T-cell receptor (TCR), and the development of T-cell exhaustion. In fact, the TME includes both CD4+ and CD8+ T-cells with regulatory and cytotoxic functions. The T-cell activation mediated by CD3 is nonselective and contributes to the activation of suppressive or regulatory T-cells within the TME that might induce drug resistance [64]. Moreover, the persistent TCR stimulation downregulates the expression of CD3 and might reduce the response of intratumoral T-cells to BsAb activity and promotes a dysfunctional phenotype of T-cells called T-cell exhaustion [65,66]. The last possible mechanism of resistance to BsAbs is T-cell-extrinsic mechanisms that include the recruitment of immunosuppressive myeloid and/or stromal cells such as tumor-associated macrophages, myeloid-derived suppressors, and cancer-associated fibroblasts in the TME [49].

## 6. Conclusions

The treatment paradigm of DLBCL has dramatically changed over the last two decades. Epcoritamab and glofitamab are currently effective new additions to the therapeutic arsenal for patients with R/R DLBCL in the third-line setting with a manageable safety profile, notably in patients who previously received treatment with CD19-targeting CAR T-cells. To date, we have several questions concerning these drugs such as their curative potential that needs longer follow-up and treatment sequences with existing treatment such as CAR T-cells and ADCs. BsAbs could represent a new cornerstone in the management of patients with DLBCL in combination with chemotherapy and immunotherapy in earlier lines especially with logistical and financial concerns with adoptive cell therapies.

## Figures and Tables

**Table 1:** Ongoing trials of bispecific antibodies as single agent.

Study	Drugs	Phase	Patient Number	Population	Primary Endpoint
NCT04703686	Glofitamab	II	78	R/R NHL after CAR T-cell	OS
NCT05207670	Mosunetuzumab	II	345	R/R B-cell malignancies	PFS, ORR
NCT05412290	Mosunetuzumab	I	15	Consolidation after ASCT in R/R aggressive B-cell lymphoma	TEAE
NCT05633615	Mosunetuzumab or other	II	396	Consolidation following CAR T-cell for R/R DLBCL or grade IIIB FL	PFS
NCT05451810	Epcoritamab	II	80	R/R DLBCL and FL	Grade ≥3 CRS Grade ≥3 ICANS
NCT05206357	Epcoritamab	I	15	R/R aggressive mature B-cell malignancies	AEs Cmax AUC
NCT04628494 (EPCORE DLBCL-1)	Epcoritamab vs. IC chemotherapy	III	480	R/R DLBCL	OS
NCT03625037 (EPCORE NHL-1)	Epcoritamab	I/II	700	R/R B-cell lymphoma	DLTs AEs ORR CRS
NCT03888105 (ELM-2)	Odronextamab	2	512	R/R B-cell NHL	ORR
NCT02290951 (ELM-1)	Odronextamab	1	298	R/R CD20+ B-cell malignancies	AEs, DLTs, ORR

R/R: relapsed and/or refractory; NHL: non-Hodgkin lymphoma; OS: overall survival; PFS: progression-free survival; ORR: overall response rate; ASCT: autologous stem cell transplantation; TEAE: treatment-emergent adverse event; Cmax: maximum concentration; AUC: area under the curve; CAR: chimeric antigen receptor; AEs: adverse events; CRS: cytokine receptor syndrome; DLT: dose-limiting toxicity; DLBCL: diffuse large B-cell lymphoma; FL: follicular lymphoma; IC: investigator’s choice; ICANS: immune effector cell-associated syndrome.

**Table 2:** Ongoing trials of bispecific antibodies in combination.

Study	Drugs	Phase	Patient Number	Population	Primary Endpoint
NCT04914741 (COALITION)	Glofitamab + R-CHOP or R-CHP-pola	I–II	80	First-line high-risk DLCBL of high-grade B-cell lymphoma	Safety RDI
NCT05800366	Glofitamab + pola-R-CHP	II	40	First-line high-risk DLBCL	CR rate
NCT04980222	Glofitamab + R-CHOP	II	40	First-line ct-DNA high-risk DLBCL	EOT CR rate
NCT04077723	Glofitamab + RO7227166	I	420	R/R NHL	DLTs, AEs, ORR, DCR, DOR, PFS, OS, CR
NCT05335018	Glofitamab + poseltinib + lenalidomide	II	76	R/R DLBCL	ORR
NCT05169515	Glofitamab + CC-220 or CC-99282	I	112	B-cell NHL	DLTs, AEs, ORR
NCT05364424	Glofitamab + R-ICE	I	40	R/R transplant or CAR T-cell eligible DLBCL	ORR
NCT03533283	Glofitamab + atezolizumab or pola	I–II	280	R/R B-cell NHL	DLTs
NCT04408638	Glofitamab + gemox versus R-GemOx	III	270	R/R DLBCL	OS
NCT05798156 (R-Pola-Glo)	Glofitamab + pola + R	II	80	First-line aggressive LBCL ineligible for R-CHOP	12-month PFS rate
NCT05219513	Glofitamab + RO7443904	I	200	R/R B-cell NHL	DLTs AEs
NCT05171647 (SUNMO)	Mosunetuzumab + pola vs. R-GemOx	III	222	R/R aggressive B-cell NHL	PFS
NCT05169515	Mosunetuzumab + CC-220 or CC-99282	I	112	B-cell NHL	DLTs, AEs, ORR
NCT05615636	Mosunetuzumab + pola + tafa + lenalidomide	II	36	R/R B-cell NHL	ORR
NCT05672251	Mosunetuzumab + loncastuximab tesirine	II	36	R/R DLBCL	Safety, ORR
NCT05260957 (ML43165)	Mosunetuzumab + pola	II	40	Consolidation following CAR T-cell in aggressive NHL	CR rate
NCT05464329	Mosunetuzumab + DHAX or ICE	Ib	40	R/R aggressive B-cell lymphoma eligible for ASCT	TEAEs
NCT05660967 (EPCORE DLBCL-3)	Epcoritamab with or without lenalidomide	II	180	First-line DLBCL	CR rate
NCT05578976 (EPCORE DLBCL-2)	Epcoritamab + R-CHOP vs. R-CHOP	III	900	Newly diagnosed DLBCL	PFS
NCT04663347 (EPCORE NHL-2)	Epcoritamab in combination	I/II	396	B-NHL	DLTs AEs
NCT04358458	GEN3009 ± epcoritamab	I/II	182	R/R B-NHL	DLTs AEs
NCT05685173 (ATHENA-1)	Odronextamab + REGN5837	1	91	Aggressive B-cell NHL	DLTs, TEAEs, AESIs

DLBCL: diffuse large B-cell lymphoma; RDI: relative dose intensity; Pola: polatuzumab vedotin; CR: complete response; ct: circulating tumor; R/R: relapsed and/or refractory; NHL: non-Hodgkin lymphoma; EOT: end of treatment; DLTs: dose-limiting toxicities; AEs: adverse events; ORR: overall response rate; DCR: disease control rate; DOR: duration of response; PFS: progression-free survival; OS: overall survival; CAR-T: chimeric antigen receptor T-cell; gemox: gemcitabine + oxaliplatine; Pola: polatuzumab vedotin; R: rituximab; GemOx: gemcitabine + oxaliplatine; DLTs: dose-limiting toxicities; AEs: adverse events; Cmax: maximum observed concentration; IC: investigator’s choice; TEAEs: treatment-emergent adverse events; AESIs: adverse events of special interest.

## Data Availability

Not applicable.
